# Interstitial Fluid Flow Increases Hepatocellular Carcinoma Cell Invasion through CXCR4/CXCL12 and MEK/ERK Signaling

**DOI:** 10.1371/journal.pone.0142337

**Published:** 2015-11-11

**Authors:** Arpit D. Shah, Michael J. Bouchard, Adrian C. Shieh

**Affiliations:** 1 School of Biomedical Engineering, Science and Health Systems, Drexel University, Philadelphia, Pennsylvania, United States of America; 2 Department of Biochemistry and Molecular Biology, Drexel University College of Medicine, Philadelphia, Pennsylvania, United States of America; University of Navarra School of Medicine and Center for Applied Medical Research (CIMA), SPAIN

## Abstract

Hepatocellular carcinoma (HCC) is the most common form of liver cancer (~80%), and it is one of the few cancer types with rising incidence in the United States. This highly invasive cancer is very difficult to detect until its later stages, resulting in limited treatment options and low survival rates. There is a dearth of knowledge regarding the mechanisms associated with the effects of biomechanical forces such as interstitial fluid flow (IFF) on hepatocellular carcinoma invasion. We hypothesized that interstitial fluid flow enhanced hepatocellular carcinoma cell invasion through chemokine-mediated autologous chemotaxis. Utilizing a 3D *in vitro* invasion assay, we demonstrated that interstitial fluid flow promoted invasion of hepatocellular carcinoma derived cell lines. Furthermore, we showed that autologous chemotaxis influences this interstitial fluid flow-induced invasion of hepatocellular carcinoma derived cell lines via the C-X-C chemokine receptor type 4 (CXCR4)/C-X-C motif chemokine 12 (CXCL12) signaling axis. We also demonstrated that mitogen-activated protein kinase (MEK)/extracellular signal-regulated kinase (ERK) signaling affects interstitial fluid flow-induced invasion; however, this pathway was separate from CXCR4/CXCL12 signaling. This study demonstrates, for the first time, the potential role of interstitial fluid flow in hepatocellular carcinoma invasion. Uncovering the mechanisms that control hepatocellular carcinoma invasion will aid in enhancing current liver cancer therapies and provide better treatment options for patients.

## Introduction

Worldwide, hepatocellular carcinoma (HCC) is the second leading cause of cancer-related deaths with over 746,000 deaths annually [1]. In the United States, it is estimated that there will be 35,560 new cases of HCC in 2015, making it one of the few types of cancer that is still increasing in incidence at a rate of approximately 3% per year [2]. Treatment of HCC remains a challenge, with 5 year survival rates for patients with stages IIC and IVA (regional HCC) of 10% and for patients with stage IVB (distant HCC) as low as 3% [3]. Chronic hepatitis B or C virus infection, non-alcoholic fatty liver disease, alcoholism, obesity, type 2 diabetes, exposure to alfatoxins, and anabolic steroids may all play a role in the development and progression of HCC [4]. The formation of intrahepatic metastases, which occurs in 51–75% of HCC tumors, is an indicator of poor prognosis [5]. Furthermore intrahepatic metastasis can be aggressive as observed in a study of 148 patients with intrahepatic HCC (stage IVA or III tumors), nearly 86% of the patients developed extrahepatic metastases occurring most frequently in the lungs [6]. Identification of early stage HCC provides the best opportunities for effectively treating this cancer; however, even if detected early, the most successful curative treatment options are limited to resection of the diseased liver tissue or liver transplantation [7]. Unfortunately, studies have shown that HCC redevelops in more than 50% of patients with intrahepatic or extrahepatic metastases within the first year [8]. Treatments for late stage or recurring HCC are also limited; palliative treatment options include transarterial chemoembolization or pharmaceutical interventions such as Sorafenib, a kinase inhibitor which has been shown in a Phase III clinical trial of 602 patients to only improve overall survival by 12 weeks. [7, 9]. Poor outcomes have been attributed to the dearth of HCC screening in the general population, limited treatment options, and invasiveness of the cancer [10]. Therefore, a better understanding of the molecular mechanisms that affect HCC development and progression is needed to develop more effective strategies for diagnosing and treating HCC.

In recent years, many studies have emphasized the importance of the tumor microenvironment in HCC progression [11]. Factors such as chronic inflammation, liver fibrosis, and cellular activity of hepatic stellate cells have been observed to alter the liver microenvironment [12]. However, the role of mechanical forces within the HCC tumor microenvironment remains poorly understood. Within the tumor microenvironment, changes in biomechanical forces such as solid stress [13], fluid pressure [14], and fluid flow [15–18] have been shown to alter cancer progression [19, 20]. Interstitial fluid flow (IFF) is one of these altered forces in the tumor microenvironment.

High permeability of tumor-associated vasculature has been shown to alter fluid movement, likely due to changes in hydrostatic and oncotic pressure [19]. Previous studies identified that most solid tumors have increased interstitial fluid pressure [21]. Interstitial fluid pressure in a healthy liver was found to be -2.2 mmHg, while the interstitial fluid pressure in a hepatoma ranged between 0–30 mmHg [22]. The resulting increase in tumor interstitial fluid pressure leads to a steep pressure gradient between the tumor and stroma that drives elevated IFF [19, 23]. Computational models have predicted IFF velocities between 0.1–6.0 μm/s under various conditions [24]. IFF velocity in mice with VEGF_165_-expressing tumors was measured to be 0.1–0.5 μm/s, and even greater velocities (1.0–8.0 μm/s) were observed in mice with human cervical carcinoma and melanoma xenografts [14, 25]. *In vivo* IFF velocities in cervical cancer patients with pelvic lymph node metastases were measured to be between 10–55 μm/s [14]. IFF has been shown to affect various cellular processes such as differentiation [26], morphogenesis [17, 27], and protein secretion [23, 28]. In cancer, IFF can promote cancer cell invasion [29, 30], alter stromal fibroblast behavior [31], and increase matrix metalloproteinase (MMP) secretion [18]. Previous studies have revealed that cells can sense interstitial flow through glycocalyx-mediated shear-stress sensing [32], autologous-gradient formation [24], and integrin activation due to fluid-drag forces and cell-matrix adhesion [17]. In a mechanism referred to as ‘autologous chemotaxis’, IFF combines with autocrine chemoattractant secretion to form an autologous gradient that directs tumor-cell migration [24, 26, 28, 29, 31, 33]. Computational models have shown that slow-moving fluid, in combination with cell-secreted proteins, can generate a pericellular chemoattractant gradient that is sufficient to trigger a physiological response such as chemotaxis [24, 29]. Previous studies have also shown that human breast cancer cell lines exhibit increased invasion in the direction of IFF via a CCR7-dependent mechanism, while glioma cell invasion can occur through CXCR4-dependent autologous chemotaxis [28, 29]. In HCC, studies have shown that CXCR4 and CXCL12 play a pivotal role in extrahepatic metastasis [34], migration [35, 36], and patient prognosis [37]. To date, however, no studies have examined the role of interstitial flow and autologous chemotaxis (potentially via CXCR4/CXCL12) in HCC cell invasion.

We hypothesized that HCC cells invade in response to IFF through the autologous formation of a CXCL12 chemokine gradient. In the studies described here, we have analyzed the effects of IFF on HCC cell invasion and have identified a key molecular pathway that is involved in liver cancer cell invasion. We show that IFF-induced HCC invasion is dependent on autologous chemotaxis via CXCR4 and CXCL12, as well as MEK/ERK activation. The results of our studies suggest a potential role for IFF in the invasive potential of HCC.

## Materials and Methods

### Cell Isolation and Culture

Hepatoma-derived Huh7 and Hep3B cells were cultured in Roswell Park Memorial Institute (RPMI) 1640 with L-glutamine (Cellgro, Manassas, VA) supplemented with 10% fetal bovine serum (FBS) and 1% penicillin/streptomycin. HepG2 hepatoblastoma-derived cells were cultured in Minimal Essential Medium (MEM) (Cellgro) supplemented with 10% FBS, 1% non-essential amino acids (NEAA), 1% sodium pyruvate, and 1% penicillin/streptomycin on collagen-coated cell-culture plates. Primary rat hepatocytes (PRHs) were isolated from 5–7 week old male Sprague-Dawley rats using a 2-step perfusion method [38]. The rat liver was perfused with 1X Liver Perfusion Buffer (Life Technologies) for 10 minutes. Next the rat liver is perfused with collagenase buffer comprised of 0.5 μM CaCl2 and 23 μg/ml Liberase Blendzyme (Roche) for 5 minutes at a flow rate of 30 ml/min. Upon completion of the perfusion, the rat liver was removed and homogenized. The homogenized rat liver was filtered through a cheese cloth and the primary rat hepatocytes were isolated via multiple centrifugations. Surgery and isolation of rat hepatocytes were approved by the Institutional Animal Care and Use Committee of Drexel University College of Medicine and complied with the Animal Welfare Act, the Public Health Service Policy of Humane Care and Use of Laboratory Animals, and the National Institutes of Health (United States) Guide for the Care and Use of Laboratory Animals [39, 40]. PRHs were used within 3 hours of isolation to prevent hepatocyte de-differentiation. The PRHs were maintained in Williams E medium (Life Technologies, Carlsbad, CA) supplemented with 2 mM L-glutamine, 1 mM sodium pyruvate, 4 μg/ml insulin, 2.2 μg/ml transferrin, 2.7 ng/ml selenium (0.4X ITS, Life Technologies), 5 μg/ml hydrocortisone, 5 ng/ml epidermal growth factor, 10 μg/ml gentamycin, and 2% dimethyl sulfoxide. All cells were maintained in a humidified 37°C environment with 5% CO_2_.

### 3D Interstitial Flow Invasion Assay

The liver cells (5.0 x 10^5^ cells/ml gel) were encapsulated in a gel comprised of 1.3 mg/ml rat tail tendon type I collagen (BD Biosciences, San Jose, CA) and 1 mg/ml Matrigel (BD Biosciences) and seeded into 12 mm diameter, 8 μm pore diameter culture inserts (Millipore, Bellerica, MA). Static and flow conditions were generated over the course of 24 hours by following a previously described method [29, 31, 41]. For the static condition, the level of basal medium outside of the transwell was level with the medium inside the transwell, resulting in little to no hydrostatic pressure difference. The IFF condition was created by adding more basal medium to the inside of that transwell than the outside, resulting in a hydrostatic pressure difference of approximately 1 mm Hg. This drove IFF through the collagen gel, with an average velocity of approximately 0.05–0.1 μm/s (based on average volumetric flow rate and the cross-sectional area of the gel). This velocity is on the lower end of velocities measured in or modeled for tumors, but much higher than the IFF velocities predicted for normal hepatic lobules [14, 25, 42]. Cells that transmigrated through the porous membrane were fixed with 4% paraformaldehyde (Sigma-Aldrich, St. Louis, MO) and stained with 4’,6-diamidino-2-phenylindole (DAPI) (2 μg/ml, MP Biomedicals, Santa Ana, CA). Fixed and stained cells were imaged by fluorescence microscopy. Both static and flow conditions were analyzed by counting five locations on the fixed membranes. Percent invasion was calculated with the following equation:

$$\%\;Invasion=\left[\frac{\left(Average\;cell\;count\right)*\left(Membrane\;surface\;area\right)}{\left(Image\;area\right)*\left(Number\;of\;cells\;seeded\right)}\right]*100$$

For specific experiments, pharmacological inhibitors, neutralizing antibodies, or recombinant proteins were added to the cell/gel mixture before gel polymerization and in the media used for the experiment at their respective target concentrations (Table 1).

**Table 1:** List of inhibitors and neutralizing antibodies.

Agent	Source	Target	Concentration	References
AMD3100	R&D Systems	CXCR4	12.6 μM	[43]
CXCL12 neutralizing antibody	R&D Systems	CXCL12	3 μg/ml	[44]
U0126	Selleckchem	MEK1/2	25 μM	[45, 46]
FR180204	Tocris	ERK1/2	10 μM	[47]

### Western Blot

Western blot was used to measure changes in total and phosphorylated protein levels in response to IFF and specific inhibitors. Cells were lysed with radioimmunoprecipitation assay (RIPA) buffer containing Halt protease and phosphatase inhibitor cocktail (Thermo Scientific, Waltham, MA). For 3D samples, cells were first digested out of the gels with collagenase D (0.5625 U/ml, Roche Life Science, Indianapolis, IN) for 45 minutes and then centrifuged for 10 minutes at 1,000 RPM. The following primary antibodies were used to detect the proteins of interest: anti-CXCR4 (1:1,000, Abcam, Cambridge, England), MEK1/2 (1:1,000, Cell Signaling Technology, Beverly, MA), phospho-MEK1/2 (Ser217/221) (1:1,000, Cell Signaling Technology, Beverly, MA), ERK1/2 (1:1,000, Cell Signaling Technology, Beverly, MA), phospho-ERK1/2 (Thr202/Tyr204) (1:1,000, Cell Signaling Technology, Beverly, MA), and β-actin (13E5) (1:2,000, Cell Signaling Technology, Beverly, MA). The following secondary antibodies were used: rabbit anti-mouse IgG-HRP (1:10,000, Abcam, Cambridge, England), goat anti-rabbit IgG-HRP (1:10,000, Abcam, Cambridge, England), and anti-rabbit IgG (1:2,000, Cell Signaling Technology, Beverly, MA). Chemiluminescence imaging was conducted with a FluorChem M imaging system (Proteinsimple, San Jose, California) and quantified with AlphaView (Proteinsimple, San Jose, California). Target protein levels were normalized to β-actin.

### CXCL12 Chemotaxis Assay

Huh7 cells (1.25 x 10^5^ cells/ml gel) were added to the same type of gel and cell-culture inserts used for the 3D flow invasion assay. Cells were exposed to a gradient of recombinant human CXCL12 in a checkerboard method, by adding a known concentration of CXCL12 (10 nM) in the medium only above, below, or above and below the gel (or a control condition with no CXCL12) for 24 hours at 37°C with 5% CO_2_. Cells that migrated through the membrane of the cell-culture insert were fixed and stained. Invasion was quantified following the same method described for the 3D flow invasion assay.

### Enzyme-linked immunosorbent assay (ELISA)

Protein from 2D cell lysates and medium were collected from approximately 8.0–11 x 10^6^ HCC cells after 4 days of incubation at 37°C and 5% CO_2_ in a T75 flask with full media. Protein from 3D cell lysates and medium were collected from 5.0 x 10^5^ liver cells after 1 day of incubation at 37°C and 5% CO_2_ in a collagen/Matrigel matrix. Protease inhibitors were added to all cell lysates and media. CXCL12 levels were quantified using a commercially available DuoSet ELISA kit (R&D Systems, Minneapolis, MN). Standard curves were generated using known concentrations of recombinant human CXCL12 and data were fit with a four parameter logistic curve in MATLAB (MathWorks, Natick, MA).

### Statistical Analysis

All data are presented as mean ± standard error of the mean (SEM). All results for invasion assays are based on a minimum of two independent experiments with sample size *n ≥* 3 for each experiment. Statistical significance between two groups was determined by conducting a Student’s t-test. For three or more groups, one or two-factor analysis of variance (ANOVA) with a Bonferroni or Tukey’s multiple comparison test was utilized. GraphPad Prism 5 (San Diego, CA) was used to perform statistical analyses.

## Results

### IFF enhances hepatocellular carcinoma cell invasion

**Fig 1:**
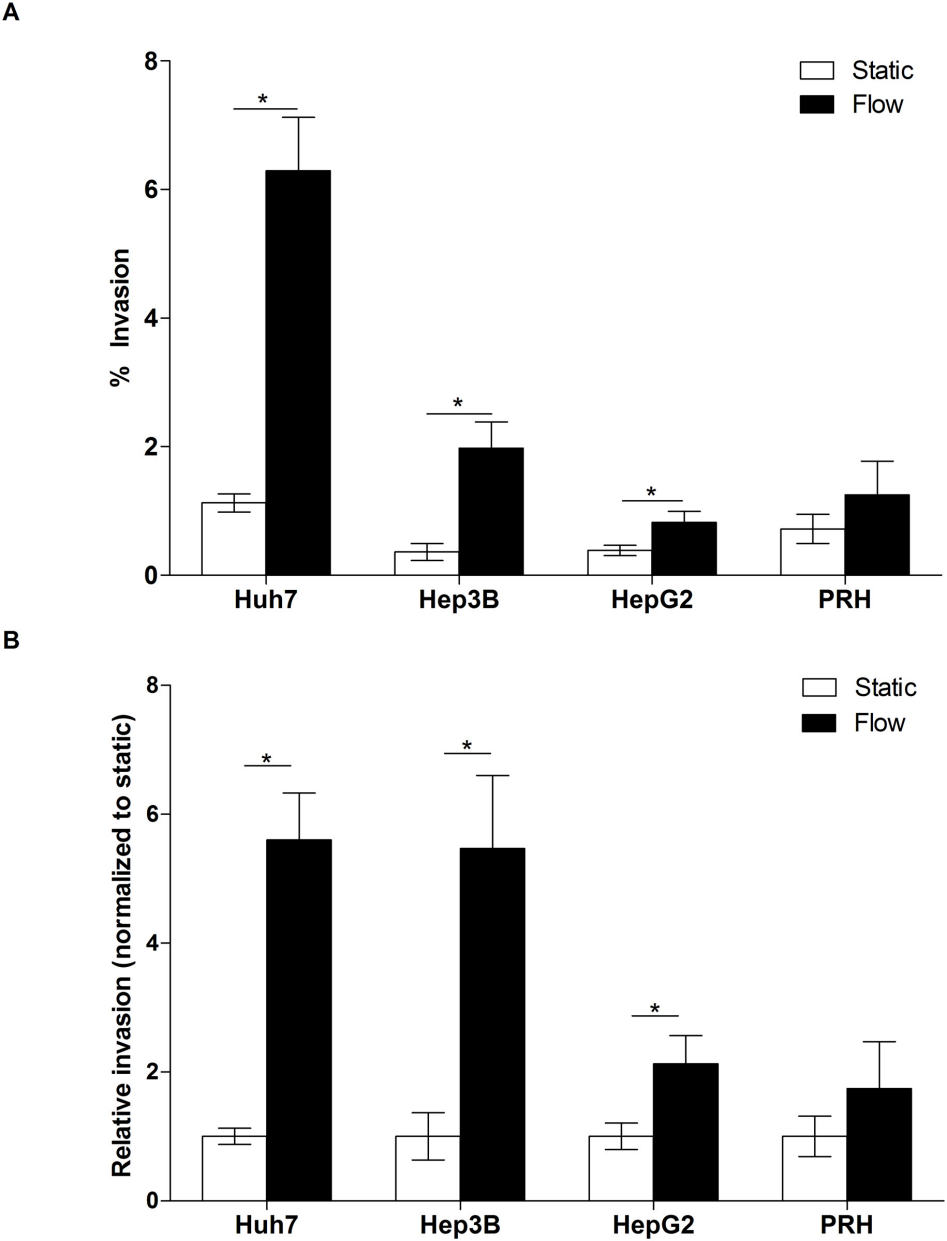
Interstitial flow induces invasion of HCC cell lines. (A) Percentage of HCC and PRHs that have invaded in response to fluid flow. Huh7, n = 23 (static and flow); Hep3B, n = 15/21 (static/flow); HepG2, n = 21/19 (static/flow); PRH, n = 9 (static and flow). *, *p* < 0.05. (B) Invasion results (identical to A) presented as normalized to each cell type’s respective static condition.

A panel of human liver-derived cell lines consisting of Huh7 (human hepatoma cells), HepG2 (human hepatoblastoma cells), and Hep3B (human hepatoma cells with integrated hepatitis B virus genome), along with PRHs, were exposed to IFF in the 3D flow invasion assay to quantify the effects of IFF on cell invasion. Huh7, Hep3B, and HepG2 cells exposed to IFF showed increased invasion in comparison to cells in static (no IFF) conditions (Fig 1A). Notably, the percent invasion of Huh7 cells exposed to IFF was much higher than Hep3B, HepG2, and PRHs (Fig 1A). When data were normalized to their respective static controls, the hepatoma-derived Huh7 and Hep3B cells showed the most dramatic response to interstitial flow, with nearly a 5.5 fold increase in invasion due to IFF (Fig 1B). In contrast, HepG2 cells showed only a 2.1 fold increase in cellular invasion in response to IFF (Fig 1B). PRHs exposed to IFF did not show any significant changes in invasion.

### IFF-induced hepatocellular carcinoma invasion depends on CXCR4

**Fig 2:**
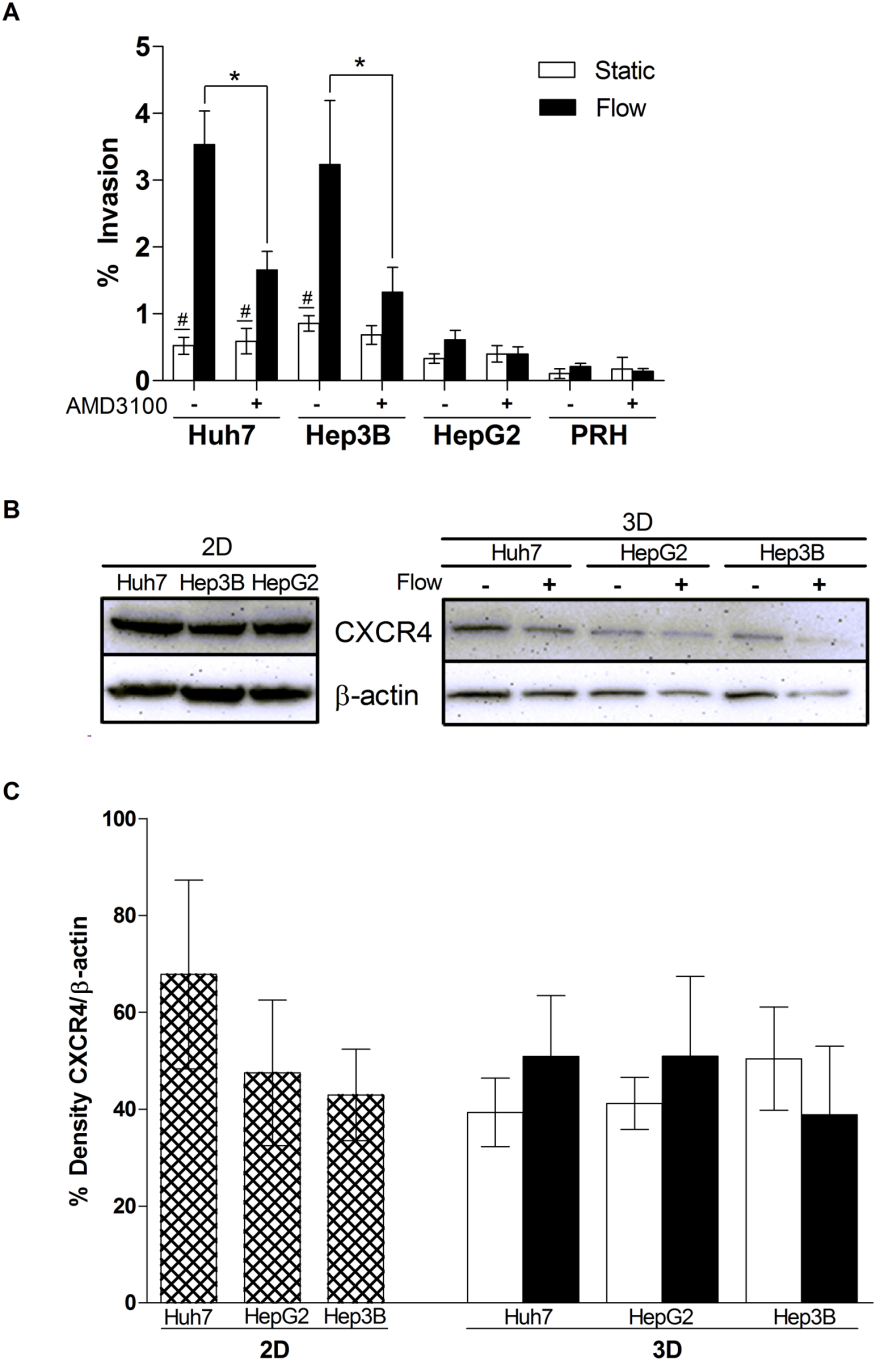
Interstitial flow-induced HCC invasion depends on CXCR4. (A) CXCR4 antagonist AMD3100 (12.6 μM) inhibits IFF-induced invasion. Huh7 (n = 18), Hep3B (n = 18), HepG2 (n = 12), and PRH (n = 6). * = *p < 0*.*05* between each respective cell line and its corresponding treatment condition. # = *p < 0*.*05* between static vs. flow conditions. (B) CXCR4 is detected in HCC cell lines in both 2D and 3D lysates. CXCR4 = 43 kDa. Static 3D sample, (-) and Flow 3D Sample, (+). (C) Quantitative western blot analysis of CXCR4 compared to its respective control, β-actin. Percentage adjusted relative density compared to loading control of respective sample. Static 3D sample, (white bar); Flow 3D Sample, (black bar). For 2D lysates (n = 3) and 3D lysates: Huh7 (n = 5), HepG2 (n = 6), and Hep3B (n = 3). No statistical significance was observed between static and flow conditions of respective cell lines.

The chemokine receptor CXCR4 has been shown to mediate cellular functions such as proliferation, migration, invasion, and adhesion in a variety of cancer types [28, 34, 48]. Studies have shown that HCC cells possess higher CXCR4 and CXCL12 protein levels in HCC metastases compared to normal hepatic tissues [34, 35]. This evidence further suggested CXCR4/CXCL12 could potentially promote tumor cell migration. Additionally, IFF has been shown to enhance glioma invasion through CXCR4/CXCL12-dependent autologous chemotaxis [28]. Thus we hypothesized that IFF-induced HCC cell invasion depends on the CXCR4/CXCL12 chemokine axis. To block CXCR4 activity, we incorporated the CXCR4 antagonist AMD3100 (12.6 μM) into the 3D invasion assay. AMD3100 significantly decreased IFF-induced invasion of both Huh7 and Hep3B cells, but had no statistical effect on HepG2 cells and PRHs (Fig 2A). To determine if the differences in IFF-induced invasion between Huh7, Hep3B, and HepG2 cells, and their sensitivity to AMD3100, was due to CXCR4 expression, we performed western blot analyses to measure CXCR4 levels. We confirmed the presence of CXCR4 in both 2D and 3D cultures of all three cell lines (Fig 2B). A quantitative analysis of CXCR4 levels showed that CXCR4 expression did not vary significantly between the cell lines, and IFF had no statistically significant effect on CXCR4 levels in 3D conditions (Fig 2C). Overall, these findings suggest that CXCR4 is necessary but not solely responsible for IFF invasion. Moreover, IFF does not modulate levels of CXCR4, nor does responsiveness to IFF correlate with CXCR4 protein expression in the cell lines tested.

### IFF-induced Huh7 invasion requires an autologous CXCL12 gradient

**Fig 3:**
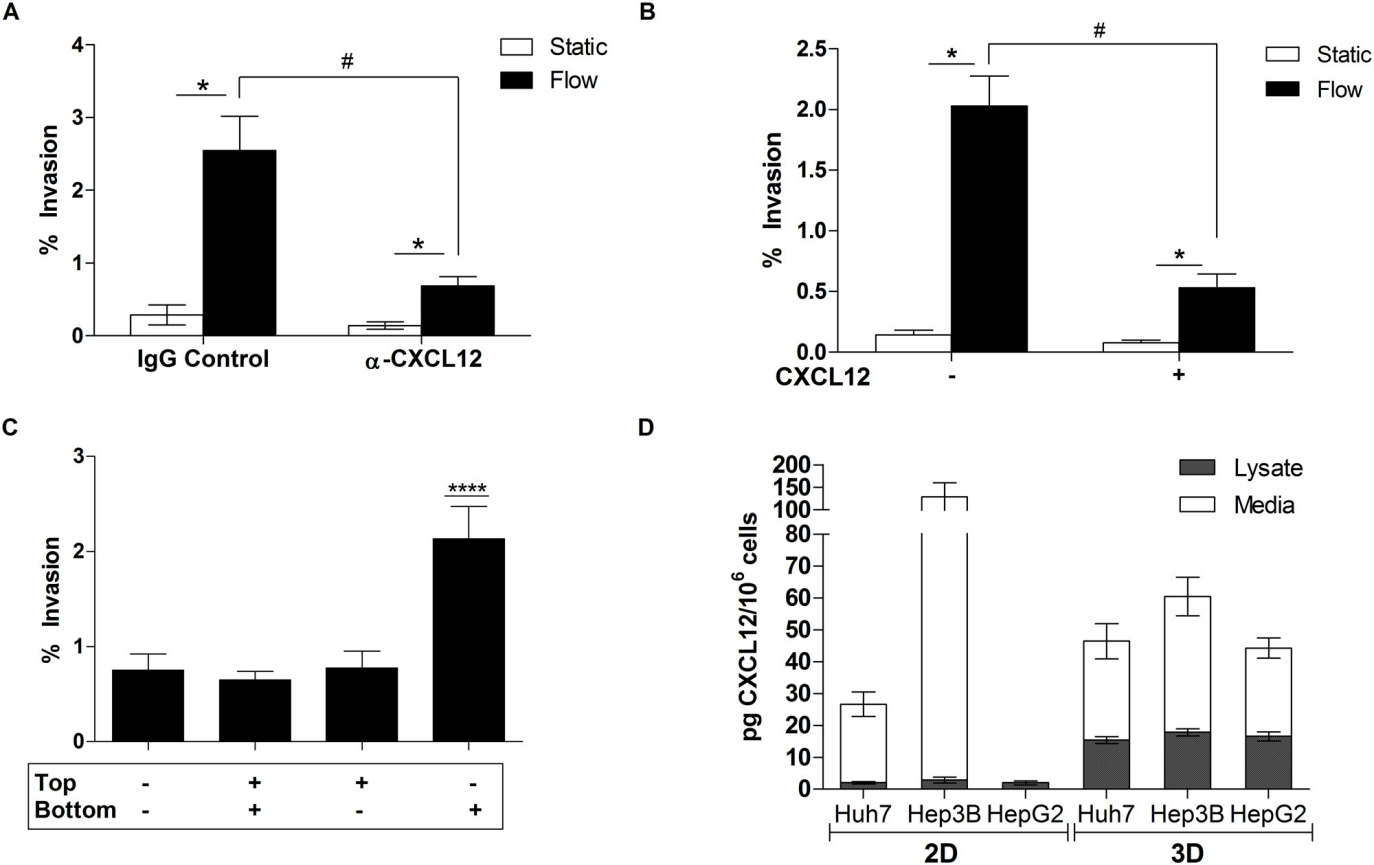
Detection of CXCL12 and its functional role in HCC. (A) Huh7 cells treated with CXCL12 neutralizing antibody and respective control (3 μg/ml) for 24 hours in a 3D invasion assay (n = 9). * = *p* < *0*.*05* between each respective treatment condition. # = *p* < 0.05 between static vs. flow conditions of the two treatment. (B) Invasion assay (n = 12) on Huh7 cells with/without CXCL12 (80 ng/ml) conditioned medium was conducted to observe changes in flow-induced cellular invasion. Test condition: + = exogenous CXCL12 added to media;—no exogenous CXCL12 present in media. Significance between static vs. flow, * = p < 0.05. # = *p* < 0.05 between static vs. flow conditions of the two treatment options. (C) Chemotaxis assay conducted on Huh7 cells in static environment. Exogenous CXCL12—80ng/ml. Test condition: + = exogenous CXCL12 added;— = no exogenous CXCL12 present. **** = *p* < 0.0001. (D) Average CXCL12 expression in 2D and 3D lysates (static) and respective medium was measured with ELISA. Sample type: cell line (n = lysate/n = medium)– 2D: Huh7 (n = 7/n = 6), Hep3B (n = 3), HepG2 (n = 3). 3D: Huh7 (n = 6), Hep3B (n = 6/n = 5), HepG2 (n = 5/n = 6).

Given the functional role of CXCR4 (Fig 2), we next investigated the involvement of CXCL12 in IFF-induced invasion of Huh7 cells. The Huh7 cells showed the strongest invasion response to IFF; therefore, we proceeded with primarily this cell line in subsequent experiments. Incorporating a CXCL12 neutralizing antibody in the 3D invasion assay resulted in a significant reduction in IFF-induced Huh7 invasion (Fig 3A), suggesting that CXCL12 is necessary for IFF-induced invasion. Based on the functional response of the Huh7 cells in our serum-free invasion assay conditions, it appeared that these cells are secreting CXCL12. One of the key features of the hypothetical autologous chemotaxis mechanism is a pericellular chemoattractant gradient caused by IFF. To diminish the relative magnitude of any cell- and IFF-generated CXCL12 gradient, we added a uniform 10 nM concentration of recombinant CXCL12 to the 3D invasion assay. This resulted in decreased IFF-induced invasion compared to the untreated condition (Fig 3B). This suggests that a CXCL12 gradient is necessary for IFF-induced invasion, consistent with the previously hypothesized autologous chemotaxis mechanism [28]. We also confirmed that Huh7 cells chemotact in response to CXCL12 through a 3D chemotaxis assay (Fig 3C). Finally, to determine if CXCL12 secretion levels vary between the Huh7, Hep3B, and HepG2 cells, which could potentially explain the differential responses to IFF, intracellular and secreted CXCL12 in 2D and 3D (static) environments was quantified by ELISA (Fig 3D). In the 2D conditions, the Hep3B cells secreted the greatest amount of CXCL12 in the medium, while HepG2 secreted no detectable amounts. The lysates of the 2D conditions exhibited low amounts of CXCL12 (~2 pg CXCL12/10^6^ cells) for all three cell lines. Between the three cell lines in 3D conditions, all three cell lines showed relatively similar levels of CXCL12 in lysate and medium. While greater amounts of CXCL12 were measured in the lysates of the cells in the 3D condition compared to the 2D condition, there was no clear correlation between IFF-induced invasion and levels of CXCL12 secretion.

### MEK/ERK signaling is required for IFF-induced hepatocellular carcinoma cell invasion

**Fig 4:**
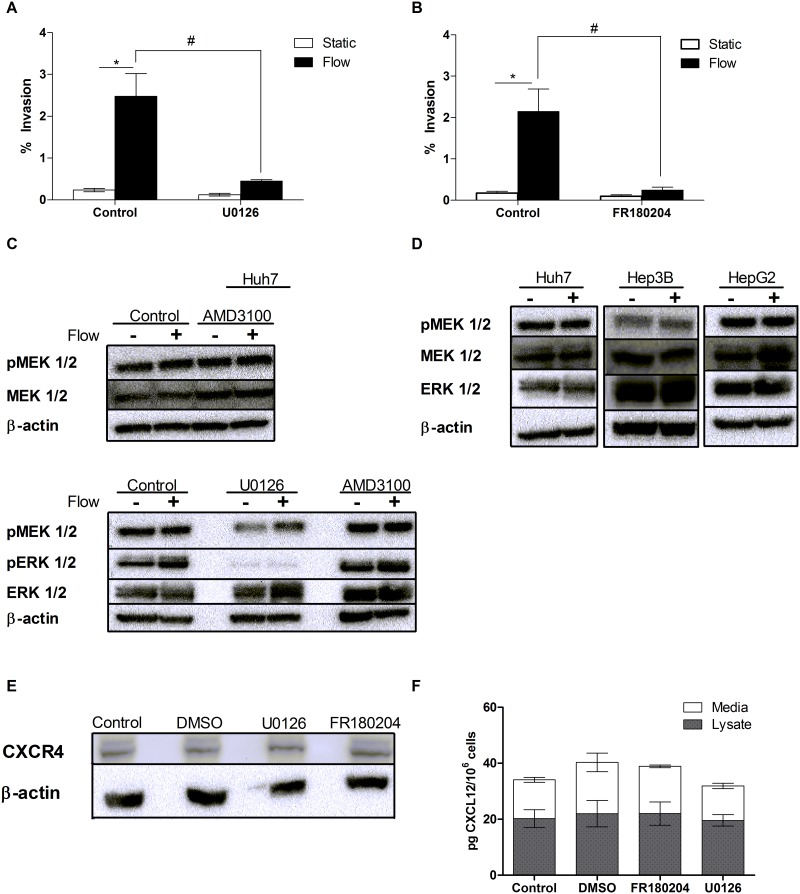
MEK/ERK required for flow-induced HCC cell invasion. (A) MEK1/2 inhibitor (U0126) at 25 μM incorporated in the 3D invasion assay with Huh7 cells (n = 14). Significance between static vs. flow, * = p < 0.05. # = *p* < 0.05 between static vs. flow conditions of the two treatment options assessed by a two-way ANOVA. (B) ERK1/2 inhibitor (FR180204) at 10 μM incorporated in the 3D invasion assay with Huh7 cells (n = 9). Significance between static vs. flow, * = p < 0.05. # = *p* < 0.05 between static vs. flow conditions of the two treatment options assessed by a two-way ANOVA. (C) Western blot conducted to detect the presence of total MEK1/2 protein collected from Huh7 cells under static and flow. pMEK1/2 was detected in Huh7 cells that were incorporated in the 3D invasion assay and exposed to AMD3100. MEK1/2 and pMEK1/2 = 45kDa. pERK1/2 was detected in Huh7 cells that were incorporated in the 3D invasion assay and exposed to treatments of U0126 or AMD3100. pERK1/2 = 42/44kDa. (D) pMEK was detected in Huh7, Hep3B, and HepG2 cells. (E) Western blot conducted to detect total CXCR4 protein collected from Huh7 cells in 3D collagen gels treated with U0126, FR180204, and DMSO (vehicle control). (F) Average CXCL12 expression measured from protein of Huh7 cells seeded in 3D collage gel and treated with U0126, FR180204, and DMSO (vehicle control).

Our previous results demonstrate that the CXCR4/CXCL12 signaling axis plays a critical role in IFF-induced HCC invasion, but utilization of a CXCR4 antagonist and the CXCL12 neutralizing antibody does not eliminate this flow-induced invasion entirely (Figs 2 and 3). Rather these results suggest that IFF-induced invasion of HCC cells is not regulated solely by CXCR4/CXCL12 chemokine signaling, but may involve other molecular mediators. Previous studies have observed elevated levels of MEK and ERK in HCC, which have been shown to contribute to HCC cell proliferation, differentiation, tumor progression, alter cell cycle regulation and apoptosis [49–51]. Given that increased MEK1/2 and ERK1/2 activity has been observed in HCC, we investigated whether these kinases affect IFF-induced HCC invasion [49, 50]. Huh7 cells in the 3D invasion assay were treated with the MEK1/2 inhibitor U0126 (25 μM), which resulted in a significant decrease in IFF-induced invasion compared to the vehicle (DMSO) control (Fig 4A). Similarly, inhibition of ERK1/2 activity by FR180204 (10 μM) resulted in significantly decreased IFF-induced invasion of Huh7 cells (Fig 4B). The inhibitor concentrations used in these experiments were confirmed to be non-cytotoxic in 3D culture conditions (S1 Fig). A western blot was conducted on protein from Huh7 3D samples and showed no change in total MEK1/2 levels between the static control and IFF condition (Fig 4C). Exposure to IFF and/or AMD3100 had no effect on phosphorylated MEK1/2 (pMEK1/2) or ERK1/2 (pERK1/2) levels in Huh7 cells (Fig 4C). This lack of change in pMEK1/2 and pERK1/2 levels with IFF or AMD3100 exposure suggests that IFF is not activating MEK or ERK via CXCR4 signaling. We also verified that U0126 inhibits MEK1/2 activity by observing the loss of pERK1/2 with U0126 treatment (Fig 4C). Levels of pMEK1/2 were similar between Huh7, Hep3B, and HepG2 cells, and IFF did not show any effect on these levels compared to their respective static conditions (Fig 4D). Furthermore CXCR4 levels were measured from the lysates of Huh7 cells seeded in a collagen gel with U0126 (25 μM) and FR180204 (10 μM) inhibitor treatments, and DMSO vehicle control (Fig 4E). No change in CXCR4 levels in the Huh7 cell was observed with MEK1/2 or ERK1/2 inhibition (S2 Fig). Similarly, there were no significant changes in secreted or intracellular CXCL12 in Huh7 cells treated with the MEK1/2 or ERK1/2 inhibitors (Fig 4F). These findings suggest that MEK/ERK signaling is involved in IFF-induced invasion, but is not upstream or downstream of CXCR4 and CXCL12. Instead, MEK/ERK may be modulating other pathways necessary for IFF-induced invasion.

## Discussion

Interstitial fluid flow is slow moving fluid flow that occurs in normal tissues; however, in tumors this fluid flow is elevated, potentially driving increased cell invasion [17, 26, 28, 31, 33]. To date, several studies have demonstrated that IFF can drive glioma, melanoma, renal, and breast cancer cell invasion [17, 28, 29, 31, 33, 52] via one of three proposed mechanisms: IFF-induced tension transduced by cell-matrix adhesions, glycocalyx-mediated shear stress sensing and downstream upregulation of matrix metalloproteinases, and autologous chemotaxis. In this study, we demonstrate for the first time that IFF can induce invasion of hepatocellular carcinoma cells through the formation of autologous transcellular gradients of CXCL12. Furthermore, IFF-induced invasion of Huh7 cells requires MEK/ERK activity independent of CXCR4/CXCL12 signaling. High expression of CXCR4 and CXCL12 has been observed in various carcinomas, resulting in increased cell migration and tumor angiogenesis [48, 53]. In hepatocellular carcinoma, CXCR4 and CXCL12 have been associated with variations in the cell cycle resulting in increased risk of metastasis formation in bone, and elevated levels of the chemokine enhances migration of the tumor cells [34, 36, 37]. We showed that HCC flow-induced invasion occurs through a CXCR4-dependent mechanism (Fig 2). Tumor dissemination and poor HCC prognosis have been linked to the expression of this chemokine receptor [35]. This CXCR4-dependent mechanism is not complete without the secretion of its ligand CXCL12. CXCL12, also known as stromal cell-derived factor 1 (SDF-1), has been shown to mediate important cellular processes such as chemotaxis and leukocyte trafficking [54]. We observed that CXCR4 and CXCL12 are necessary for IFF-induced invasion and that the levels of the chemokines did not change when exposed to IFF (Fig 3). The presence of CXCR4 and CXCL12 was necessary for IFF-induced invasion in HCC cells. A potential reason as to why non-HCC cells such as the PRHs did not respond to flow-induced invasion could be due to the lack or low levels of either the CXCR4 receptor or its ligand CXCL12. Previous work identified CXCR4 messenger RNA (mRNA) and protein levels in HCC tissues and cell lines, but could not detect CXCR4 in normal hepatic tissues. Similarly, CXCL12 was detectable in HCC patients’ ascites fluid but not in normal hepatic tissue [34]. No further experiments were conducted with PRHs because they did not respond to IFF; instead, we focused our efforts to uncover the molecular mechanisms of IFF-induced HCC cell invasion. Ultimately, we were unable to identify any relationship between total protein levels of CXCR4 or CXCL12 and response to IFF; however it is important to note that autologous chemotaxis does not require increased expression of the receptor or secretion of the ligand, but requires the formation of a gradient [24]. Furthermore we observed differences in IFF-induced invasion between the three liver cancer cell lines in our study. It has been observed that Huh7 and Hep3B cells, exposed to CXCL12, respond with rapid perinuclear translocation of CXCR4 while HepG2 cells do not due to a receptor defect [35, 55]. Moreover, it was also previously shown that Huh7 cells showed a strong invasive response once exposed to CXCL12, which is quite similar to our findings (Fig 3) [35]. In conclusion, we identified the CXCR4/CXCL12 signaling axis to be a significant component in IFF-induced invasion in HCC; however, this signaling was not the only mechanism involved in IFF-induced invasion of HCC cells, as shown by the presence of some IFF-induced invasion even with inhibition of CXCR4 or CXCL12 (Figs 2A and 3B).

In the past twenty years, much emphasis has been placed on the RAF-MEK-ERK signaling cascade to better understand its potential therapeutic benefits for cancer therapy. The MEK/ERK signaling cascade has been observed to be highly active in HCC and shown to regulate invasion and formation of metastasis in HCC cells [49]. Recent studies investigating the molecular pathogenesis of HCC have revealed that blocking MEK/ERK signaling in HCC cells results in multiple anticancer effects such as decreased HCC cell proliferation, growth, and increased apoptosis [51]. In HCC tissue specimens, MEK/ERK signaling has been shown to be constitutively activated in many specimens, and inhibition of the MEK/ERK pathway in our study resulted in a significant decrease of IFF-induced invasion (Fig 4A and 4B) [49]. Some studies have also suggested crosstalk between CXCR4/CXCL12 activation and MEK/ERK signaling that influences cellular invasion [56]. One study demonstrated that CXCR4/CXCL12 signaling resulted in increased phosphorylation of ERK in Huh7 cells [43]. In contrast, we demonstrated that CXCR4/CXCL12 signaling did not alter the MEK/ERK pathway in our liver cell lines (Fig 4C). This could be a result of the differences in the experimental model; our studies are performed in 3D, while the previous study used cells cultured in 2D. Furthermore, we determined that MEK/ERK signaling and chemokine signaling independently altered HCC cell invasion (Fig 4E and 4F). Previous studies have observed MEK/ERK activity to be constitutively elevated in HCC as well as other carcinomas, altering many cell functions such as proliferation, differentiation, and apoptosis [49, 56]. We propose that MEK and ERK signaling affect one or more cell functions that are essential for IFF-induced invasion; however this signaling cascade is working independently from CXCR4/CXCL12 signaling axis. We hypothesize that this high constitutively active MEK and ERK signaling are essential mediators for flow-induced invasion and could be affecting a different aspect of the invasion process such as cellular adhesion, contractility, and matrix remodeling [57–59]. The MEK/ERK signaling cascade has been implicated in the enhanced secretion of MMPs in HCC resulting in increased cell migration and invasiveness [60, 61]. Activation of the MEK/ERK pathway along with cell surface receptors have been shown to mediate invasion [44, 62]. However further investigation would be required to elucidate the role of MEK/ERK signaling in IFF-induced invasion.

In this study, we elucidated CXCR4/CXCL12-dependent autologous chemotaxis as a significant mechanism involved in IFF-induced invasion of HCC cells. Additionally we identified MEK/ERK signaling as a significant contributor to IFF-induced invasion, but one that is independent of the CXCR4/CXCL12-autologous chemotaxis mechanism. However as previously mentioned, mechanisms of interstitial fluid flow mechanosensing are not fully understood. The formation of autologous transcellular gradients is merely one potential mechanism that cells may use to sense and respond to IFF. Another possible mechanism involves glycocalyx shear stress sensing, where flow-induced stresses are transduced into and transmitted as solid stresses through core proteins of the glycocalyx, leading to various intracellular signaling cascades [15–17, 32]. These studies identified that IFF mechanotransduction occurs through glycocalyx heparan sulfate proteoglycans and focal adhesion FAK-ERK signaling, resulting in increased cell motility through increased MMP activity in 3D collagen gels [63]. Focal adhesion kinase (FAK) signaling to mitogen-activated protein kinase (MAPK) has been observed to be involved in migration and differentiation in HCC [57]. It may be possible that glycocalyx-mediated IFF sensing in HCC could occur through signaling of FAK to MAPK activation resulting in increased cell motility. This may be a potential mechanism since we observed MEK/ERK signaling, an important constituent in IFF-induced invasion in HCC, but could not identify any relationship to our CXCR4/CXCL12 results. Additionally we observed CXCR4 inhibition with AMD3100 did not completely eliminate HCC-flow induced invasion (Fig 2A). CXCL12 could also act through an auxiliary receptor such as syndecan-4 (SDC-4), a transmembrane heparan sulfate proteoglycan that has been shown to regulate the migration response of HCC cells to CXCL12 gradients [43]. Alternatively, IFF could also induce invasion through mechanisms entirely independent of CXCR4/CXCL12. Previous studies have shown that IFF stimulated upstream invasion (against the direction of flow) of breast cancer cells through activation of integrins and focal adhesion reorganization and polarization [17, 30]. Given the nature of our 3D flow invasion assay and study, we only measured downstream invasion; invasion in the opposite direction would not be captured by our transwell system. Since the changes we observed resulted in increased invasion in the direction of IFF, we can also conclude that the mechanisms at play are likely separate from any induction of upstream invasion, since we are explicitly not quantifying that component of the invasion response.

In conclusion, we demonstrated that IFF is a critical factor in hepatocellular carcinoma invasion, and potentially drives invasion through two separate signaling pathways. First, we show that HCC cells invade in response to IFF via a CXCR4/CXCL12-dependent mechanism. Our findings suggest that this occurs through autologously generated pericellular gradients due to HCC cell-secreted CXCL12 and IFF, corroborating previous studies of the autologous chemotaxis mechanism [24, 28, 29, 52]. Second, we have evidence that the MEK/ERK pathway plays a critical role in IFF-induced HCC invasion, but one that is not downstream of CXCR4/CXCL12. Our study provides a better understanding of how biomechanical forces like IFF can alter signaling pathways that drive hepatocellular carcinoma invasion, and may potentially provide a basis for investigating new therapeutic strategies for HCC. Ultimately, the goal is to reduce HCC invasion and prevent HCC from spreading intraphepatically or metastasizing to other organs. We have shown some HCC cells become invasive upon exposure to this subtle fluid flow, and identifying these specific cells that respond strongly to flow could result in new therapeutic strategies to prevent invasion. Based on our study, it is evident that biomechanical forces such as IFF can affect HCC cell invasion. However, in order to better understand the role of mechanical forces within the tumor microenvironment, the development of complex *in vitro* models is required along with a greater understanding of how cellular machinery is utilized to sense and react to these micro-environmental changes.

## Supporting Information

S1 Fig Live/Dead assay on Huh7 cells. A Live/Dead assay conducted on Huh7 cells to confirm inhibitor concentrations used in experiments were non-cytotoxic in 3D culture conditions.
(TIF)

S2 Fig Quantitative western blot analysis of CXCR4 compared to β-actin. Percentage adjusted relative density compared to loading control of respective 3D static sample. Huh7 cells were treated with U0126 at 25 μM or FR180204 at 10 μM.
(TIF)
