# Supplementary figures and images for: Interstitial Fluid Flow Increases Hepatocellular Carcinoma Cell Invasion through CXCR4/CXCL12 and MEK/ERK Signaling
Source: PLoS One. 2015 Nov 11;10(11):e0142337. doi: 10.1371/journal.pone.0142337 (PMC4641731; doi:10.1371/journal.pone.0142337)

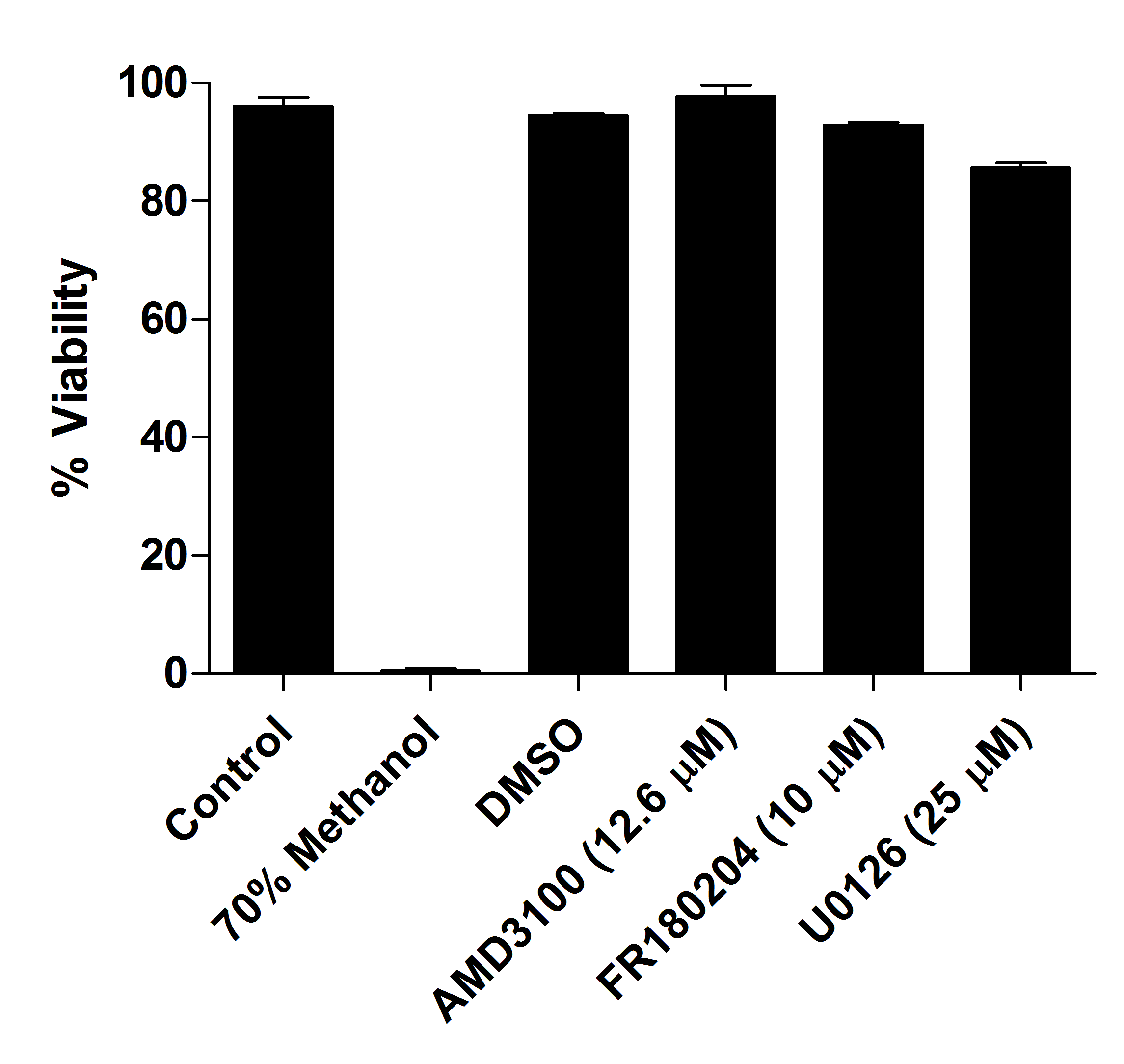

Supplement: S1 Fig — A Live/Dead assay conducted on Huh7 cells to confirm inhibitor concentrations used in experiments were non-cytotoxic in 3D culture conditions. (TIF) [file pone.0142337.s001.tif]

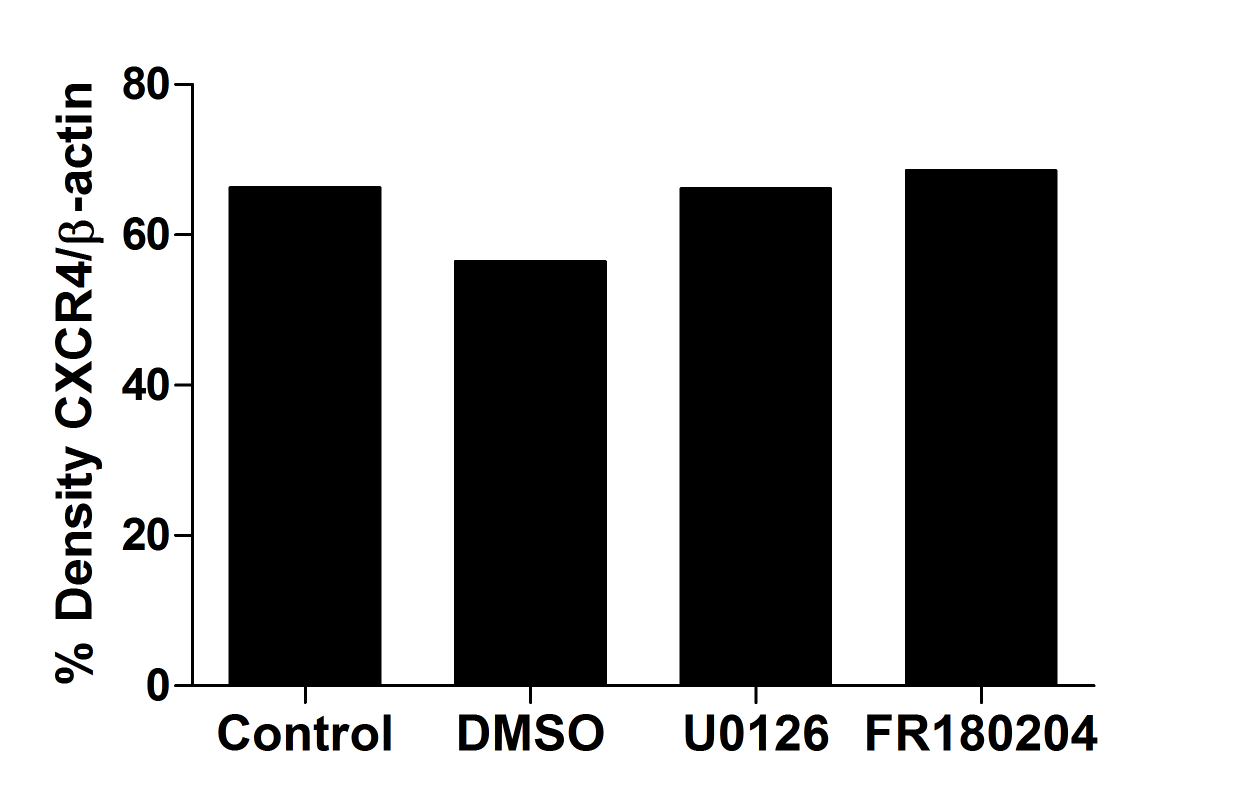

Supplement: S2 Fig — Percentage adjusted relative density compared to loading control of respective 3D static sample. Huh7 cells were treated with U0126 at 25 μM or FR180204 at 10 μM. (TIF) [file pone.0142337.s002.tif]
